# Salubrinal Exposes Anticancer Properties in Inflammatory Breast Cancer Cells by Manipulating the Endoplasmic Reticulum Stress Pathway

**DOI:** 10.3389/fonc.2021.654940

**Published:** 2021-05-20

**Authors:** Andrew Alsterda, Kumari Asha, Olivia Powrozek, Miroslava Repak, Sudeshna Goswami, Alexandra M. Dunn, Heidi C. Memmel, Neelam Sharma-Walia

**Affiliations:** ^1^ H. M. Bligh Cancer Research Laboratories, Department of Microbiology and Immunology, Chicago Medical School, Rosalind Franklin University of Medicine and Science, North Chicago, IL, United States; ^2^ Lake Forest College, Lake Forest, IL, United States; ^3^ Advocate Health Care, Park Ridge, IL, United States

**Keywords:** endoplasmic reticulum stress, inflammatory breast cancer, Salubrinal, phenylbutyrate, osteoprotegerin

## Abstract

The endoplasmic reticulum (ER) regulates protein folding, post-translational modifications, lipid synthesis, and calcium signaling to attenuate the accumulation of misfolded proteins causing ER stress and maintains cellular homeostasis. The tumor microenvironment is rich in soluble cytokines, chemokines, growth, and angiogenic factors and can drive the ER’s abnormal functioning in healthy cells. Cancer cells adapt well to the tumor microenvironment induced ER stress. We identified that the inflammatory breast cancer (IBC) cells abundantly express osteoprotegerin (OPG) and their tumor microenvironment is rich in OPG protein. OPG also called osteoclast differentiation factor/osteoclastogenesis inhibitory factor (OCIF) is a soluble decoy receptor for receptor activator of nuclear factor-kappa B ligand (RANKL). Employing mass spectrometry analysis, we identified a set of ER chaperones associated with OPG in IBC cell lysates (SUM149PT, SUM1315MO2) compared to healthy human mammary epithelial cells (HMEC). Proximity ligation assay (PLA) and immunoprecipitation assay validated the interaction between OPG and ER chaperone and master regulator of unfolded protein response (UPR) GRP78/BiP (glucose-regulated protein/Binding immunoglobulin protein). We detected remarkably high gene expression of CCAAT enhancer-binding protein homologous protein (CHOP), inositol-requiring enzyme 1 (IRE1α), protein disulfide-isomerase (PDI), PKR-like ER kinase (PERK), activating transcription factor 4 (ATF4), X-box binding protein 1 (XBP-1) and growth arrest and DNA damage-inducible protein (GADD34) in SUM149PT and SUM190PT cells when compared to HMEC. Similarly, tissue sections of human IBC expressed high levels of ER stress proteins. We evaluated cell death and apoptosis upon Salubrinal and phenylbutyrate treatment in healthy and IBC cells by caspase-3 activity and cleaved poly (ADP-ribose) polymerase (PARP) protein assay. IBC (SUM149PT and SUM190PT) cells were chemosensitive to Salubrinal treatment, possibly *via* inhibition in OPG secretion, upregulating ATF4, and CHOP, thus ultimately driving caspase-3 mediated IBC cell death. Salubrinal treatment upregulated PDI, which connects ER stress to oxidative stress. We observed increased ROS production and reduced cell proliferation of Salubrinal treated IBC cells. Treatment with antioxidants could rescue IBC cells from ROS and aborted cell proliferation. Our findings implicate that manipulating ER stress with Salubrinal may provide a safer and tailored strategy to target the growth of inflammatory and aggressive forms of breast cancer.

## Introduction

**Figure 1:**
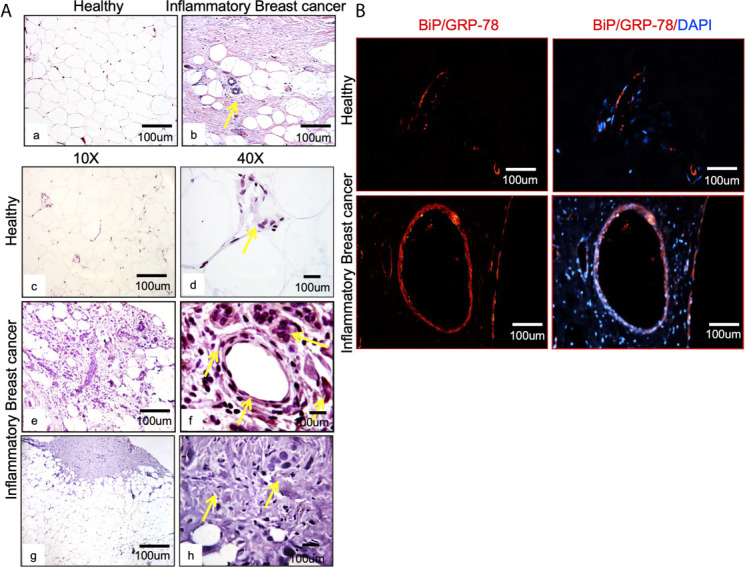
BiP/GRP78 is expressed abundantly in human IBC tissue sections. **(A)** Hematoxylin and Eosin (H & E) staining of healthy (left) **(Aa)** and IBC tissue (right) **(Ab)** is shown at 10X and 40X magnification. Healthy **(Ac, Ad)** and IBC tissue sections **(Ae, Af)** were stained using an antibody against BiP/GRP78. Human IBC tissue sections **(Ag, Ah)** were stained using an anti-IgG antibody. **(Aa–Ac, Ae, Ag)** are 10X, whereas **(Ad, Af, Ah)** represent 40X magnification. **(B)** BiP/GRP78 immunostaining (red) and healthy and IBC tissue are shown at 10X magnification. Tissue sections were developed with Alexa-594 coupled secondary antibody (red). Nuclei were visualized using DAPI as the counterstain (blue).

**Figure 2:**
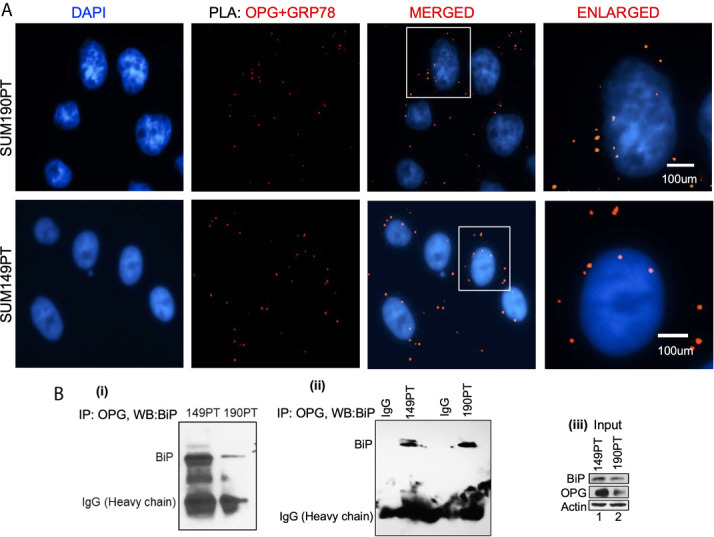
OPG interacts with BiP/GRP78 in IBC cell lines. **(A)** Proximity ligation assay (PLA) was performed using IBC cell lines, SUM149PT, and SUM190PT. Cells were fixed, permeabilized, and incubated with primary antibodies against OPG and BiP/GRP78, and then labeled with DuoLink PLA plus and minus probes. A red fluorescent probe was used to detect amplified DNA. Nuclei were visualized using DAPI as the counterstain (blue). **(B)** Association of OPG and GRP78 in IBC cell lines. Co-immunoprecipitation of OPG-BiP/GRP78 complex using anti-OPG antibody or anti-IgG antibody and visualization of the complex *via* Western blotting using anti- BiP/GRP78 antibody.

Inflammatory breast cancer (IBC), a rare but extremely invasive and aggressive disease, is characterized clinically by inflammation symptoms. Invasion of dermal lymphatics leads to tissue swelling, warmth, and tenderness, and a distinctive peau d’orange appearance of the skin. Histologically, IBC identifies as an invasive ductal carcinoma (IDC) with high-grade features, including pleomorphic cells and atypical mitotic figures (1, 2). IBC is commonly estrogen, progesterone, and HER-2 receptor-negative (triple-negative breast cancer) or TN-IBC (3). It is not vulnerable to current trimodal (chemotherapy, radiation, and surgery) (4) and there is a strong need for novel targeted therapies (5). These characteristics collectively amount to a particularly poor prognosis and the likely chemotherapy resistance development (2, 4, 6). Even though advances in treatment modalities, including neoadjuvant chemotherapy, have increased survival rates, the forecast for IBC remains significantly worse than non-inflammatory locally advanced breast cancer (7, 8). Thus, there is a critical need to advance our understanding of IBC biology and develop novel treatments (9, 10). Resistance to apoptosis is a significant problem associated with the poor prognosis of IBC. Tumor necrosis factor-related apoptosis-inducing ligand (TRAIL), a potent endogenous activator of cell death, preferentially kills transformed cells over healthy cells. Ionizing radiation can sensitize breast carcinoma cells to TRAIL-induced apoptosis in a p53-dependent manner (11). We demonstrated that IBC cell lines heavily express osteoprotegerin (OPG) and secrete it in their tumor microenvironment (12, 13). OPG induced survival, proliferation, and aneuploidy when added to the growth medium of healthy human mammary epithelial cells (HMEC) (14). OPG also plays an essential role in multiple myeloma and cancers of the prostate, bladder, and stomach (15). Expression of OPG, TRAIL, and receptor activator of nuclear factor κB ligand (RANKL) in human breast tumors has also been reported by other research groups (16–18). Interestingly, OPG binds to TRAIL and suppresses TRAIL’s function in inducing apoptosis in ameloblastomas (19, 20). OPG protects ovarian cancer cells from TRAIL-mediated apoptosis, and recombinant OPG could abrogate the antitumor effect of TRAIL and correlates with poor prognosis (20).

ER stress pathway plays an essential role in cancers of the breast, prostate, pancreas, liver, CNS, colon, and ovary (21–24). Tumor microenvironment-induced cellular stress has emerged as one of the critical factors involved in the evolution of cancers towards aggressiveness and metastatic dissemination. The ability of tumors to adapt to a hostile environment is an essential hallmark of the disease. As cancer expands, it outgrows its primary blood supply, leading to hypoxia, nutrient deprivation, oxidative stress, an acidic pH, and cell-based cancer-associated fibroblasts (CAFs), cancer-associated macrophages (CAMs), endothelial precursors induced stress mechanisms. These stressors are especially burdensome on the ER of rapidly dividing cells, which must synthesize proteins needed for growth (25–27). The ER provides a unique environment that facilitates the folding and transport of various secretory proteins. This tightly regulated environment involves coordinating many protein folding enzymes, including calcium-dependent chaperones that help proteins attain their native conformation. The ER also regulates calcium homeostasis through Ca^2+^ATPases that actively transport calcium into the ER. Besides, the oxidative environment facilitated by ER chaperones protein disulfide-isomerase (PDI) and ER oxidoreductase-1 (ERO1) promotes the formation of disulfide bonds needed for proper protein folding. Significant changes to this environment disrupt protein folding and lead to an accumulation of unfolded proteins within the ER lumen; such a state is known as ER stress.

A buildup of unfolded or misfolded proteins within the ER induces unfolded protein response (UPR), which is the first step to stop protein synthesis and allow selective ER chaperones such as binding immunoglobulin protein GRP78/BiP to restore balance. UPR can be both cytoprotective and cytotoxic. At first, the UPR works to regain protein folding, but prolonged stress may induce apoptosis. The UPR is activated when ER chaperones bind to exposed hydrophobic groups of unfolded proteins. GRP78/BiP is critical for protein quality control in the ER. GRP78/BiP is primarily responsible for activating the UPR and ER-transmembrane signaling molecules such as inositol-requiring enzyme 1 (IRE1α), PKR-like ER kinase (PERK), activating transcription factor 6 (ATF6) (28). These proteins are stress sensors that activate three corresponding signaling pathways responsible for restoring homeostasis and upregulate chaperones’ production, arresting proteins’ translation, and stimulating protein degradation (28). PERK activation also leads to ATF4 and CHOP production, a well-characterized marker for activation of the proapoptotic module of the ER stress pathway (29, 30). We identified the proteins by mass spectrometric analysis of the immunoprecipitate of anti-OPG with lysates prepared from healthy mammary epithelial cells (HMEC), IBC cell lines (SUM149PT, SUM1315MO2). We observed a strong association between OPG and cellular chaperone GRP78/BiP in IBC cells and IBC tumor sections from patients (12). OPG’s interaction with the master regulator of UPR suggested that OPG overexpression and substantial secretion from IBC cells may have a potential link in regulating ER stress or UPR in IBC (12). To understand this link, we first assessed ER stress proteins’ levels in human IBC tissues and cell lines. Having observed the abundant expression of ER stress/UPR proteins in IBC cell lines and tissue sections compared to their healthy counterparts, we chose to study the effect of Salubrinal treatment on IBC cells. Salubrinal is a synthetic cell-permeable chemical agent (phosphatase inhibitor) that can be taken as an oral pill and is known to elevate the levels of phosphorylated eukaryotic translation initiation factor 2α (eIF2α) (31–33). Salubrinal was chosen because a) its therapeutic potential has been tested in other cancers (31–33), b) its ability to target ER stress pathways (31–33), and c) Salubrinal’s enhanced activity in cells harvested from the osteoporotic bone samples in context with RANKL (34, 35). Recent studies using Salubrinal demonstrated its potential for the treatment of osteoporosis, the role of stimulation of bone formation, attenuation of bone resorption also encouraged us to study its effect in the context of highly metastatic IBC, which is strongly associated with bone loss. Salubrinal has demonstrated antitumor potential as a combination therapy with doxorubicin (36) and rapamycin (37). The specific role of ER stress in IBC biology remains unclear, and a greater understanding will promote the development of new therapeutic strategies and more favorable prognoses.

## Materials and Methods

### Agents, Cell Lines and Cell Culture

Primary human mammary epithelial cells (HMEC) (#830-05a, Cell Applications, San Diego, CA) were cultured in HMEC medium (#815-500, Cell Applications). Inflammatory breast cancer cells (SUM149PT and SUM190PT) were obtained from Asterand, Detroit, MI. All cells were tested for mycoplasma contamination by the standard Limulus assay (Limulus amebocyte lysate endochrome; Charles River Endosafe, Charleston, SC) method as per the manufacturer’s instructions. All cells were cultured in lipopolysaccharides (LPS) free medium. Antibodies against GRP78/BiP (#3183), PERK (#5683S), IRE1α (#3294S), Calnexin (#2433S), Cleaved PARP (#5625), ERO1α (#3264S), and PDI (#2446S) were obtained from Cell Signaling Technology, Danvers, MA. Antibody against β-actin (#A2228) was obtained from Sigma Aldrich, St. Louis, MO. Antibody against Caspase 3 (66470-2-Ig) was obtained from Proteintech., Rosemont, IL.

Anti-Caspase 3 Antibody, active (cleaved) form (AP1027) was obtained from Calbiochem. Salubrinal; C_21_H_17_Cl_3_N_4_OS (#CML0951), N-Acetyl-L-cysteine (#A7250) and phenylbutyrate (#SML0309) were purchased from Sigma Aldrich.

### Tissue Sections

We received 12 samples of inflammatory carcinoma, healthy breast tissue from Advocate Lutheran General Hospital under the approved IRB (IRB00001341). The Inclusion criteria were Pre- and post-menopausal women who have been diagnosed with inflammatory breast cancer *via* biopsy but have not received therapy for this disease yet. The Inclusion criteria also included the women of age 18 years or older. Exclusion criteria were 1) Patients with a psychiatric history that would prevent informed consent, 2) Patients with prior history of invasive malignancy within the last ten years, 3) Pregnant or lactating patients. Healthy breast tissue was obtained from non-cancer/healthy individuals undergoing reduction mammoplasty.

### Immunohistochemistry (IHC)

Paraffin-embedded sections (patients with IBC tumors and sections from healthy individuals) were obtained through our collaboration with Lutheran General Hospital. Sections were deparaffinized with HistoChoice clearing reagent and hydrated with water before microwave treatment in 1 mmol/liter EDTA (pH 8.0) for 15 min for antigen retrieval and then blocked with blocking solution (2% donkey serum, 0.3% Triton X-100 in phosphate-buffered saline. Cells were incubated with the primary antibody for GRP78/BiP overnight at room temperature. Slides were then washed with phosphate-buffered saline (PBS) and incubated with HRP-labeled secondary antibodies for 30 min and developed using DAB reagent (DAKO) as per methods described previously (38). IHC was also performed using IgG control antibody as described previously (38). Counterstaining was done by hematoxylin (38). Conjugates of anti-mouse/rabbit-alkaline phosphatase and anti-mouse/rabbit-horseradish peroxidase were from Kirkegaard and Perry Laboratories, Inc., Gaithersburg, MD.

### Gene Expression Analysis by Real-Time qRT-PCR

Total RNA was isolated using TRIzol Reagent (#15596026, Life Technologies Corporation, Grand Island, NY) from IBC tissue samples (Biochain, breast tumor tissue array # T22350862-2) and treated with DNase I (#18068015, Life Technologies Corporation) at 37°C for 30 min for DNA removal. RNA quantification was done using NanoDrop (Thermo Fisher Scientific). Reverse transcription was performed using the high-capacity cDNA reverse transcription kit (#4368814, Life Technologies Corporation), converting RNA to cDNA. Transcripts of the genes of interest were detected by real-time quantitative PCR using gene-specific primers. The relative abundance of target gene mRNA was calculated using the delta-delta method (ratio, 2^-[ΔCt sample-ΔCt control]^) as described previously (38). Normalization was done with respect to 18S ribosomal RNA levels.

### Proximity Ligation Assay (PLA)

SUM149PT and SUM190PT cells were fixed, permeabilized, and incubated with primary antibodies from different species against OPG and BiP/GRP78. They were then labeled with corresponding DuoLink PLA plus and minus probes as per the manufacturer’s protocol (# DUO92101Sigma Aldrich). Following ligation and amplification, amplified DNA was detected *via* the hybridization of a red fluorescent probe. Nuclei were visualized using 4′, 6-diamidino-2-phenylindole (DAPI), a blue-fluorescent DNA stain (Ex358/Em461; # P36962, Molecular Probes, Eugene, OR).

### Western Blot Analysis

Total cell lysates were prepared from respective cells in RIPA buffer containing 15 mM NaCl, 1 mM MgCl2, 1 mM MnCl2, 2 mM CaCl2, 2 mM phenyl-methylsulfonyl fluoride, and protease inhibitor mixture (Sigma). The cell lysates were centrifuged at 13,0006 g for 20 min at 4°C and protein concentration was quantified using Pierce BCA protein assay. Equal amounts of protein (20 µg/lane) were separated on SDS-PAGE, electrotransferred to 0.45-µm nitrocellulose membranes, blocked with 5% BSA, probed with specific primary antibodies (overnight at 4°C) of interest, and visualized using an enhanced chemiluminescence detection system as described previously (38). Species-specific horseradish peroxidase secondary antibodies were used for detection. Immunoreactive bands were developed by Pierce Super Signal West Pico or Femto reagents (Pierce, Rockford, IL). The bands were scanned and quantitated with ImageJ software following standard protocols (38). β-Actin was used as a loading control.

### Immunofluorescence Assay (IFA)

HMEC, SUM149PT, and SUM190PT cells were seeded in eight-well chamber slides (Nalge Nunc International, Naperville, IL.) fixed with 4% paraformaldehyde, permeabilized with 0.4% Triton X-100, and stained with primary antibody overnight at 4°C. Cells were washed and developed with Alexa 594 or Alexa 488-coupled secondary antibody (Molecular Probes), and nuclei were visualized using DAPI counterstain. Stained cells were washed and viewed with the appropriate filters on an Olympus confocal laser-scanning microscope (Fluoview FV10i) with the Metamorph digital imaging system (38–40).

### Cytotoxicity Assay

The viability of the cells after treatment with Salubrinal or phenylbutyrate was determined by lactate dehydrogenase (LDH) measuring cytotoxicity assay (#G1780) (Promega, Madison, WI), as described previously (40). LDH is released into cell culture media when the plasma membrane is damaged. The assay measures this extracellular LDH using an enzymatic reaction that results in a red formazan product, which absorbs strongly at 490 nm and can be measured spectrophotometrically. The amount of color formed is proportional to the number of lysed cells.

### Caspase-3 Activity Assay

Caspase-3 activity was measured in cell lysates prepared from untreated, or Salubrinal treated HMEC, SUM149PT, and SUM190PT cells by a Caspase-Glo-3 test from Promega (#G8091) as per the manufacturer’s instructions (40). Cells are seeded in a 96-well plate, the reagent is added, including a luminogenic caspase-3 substrate, and the luminescent signal is measured to determine the amount of caspase activity that is present.

### Cleaved Poly (ADP-Ribose) Polymerase (PARP) Protein Assay

The activity of PARP, a nuclear DNA-repair enzyme, is increased in response to DNA damage. However, PARP can activate a unique cell death program during excessive DNA damage by generating large branched ADP-ribose polymers. During apoptosis, PARP is cleaved by activated caspase-3. Following Salubrinal treatment in HMEC, SUM149PT, and SUM190PT cells, the cleaved PARP level was measured using the human cleaved PARP1 in-cell ELISA kit (Abcam Cambridge, MA) per the manufacturer’s instructions.

### Cell Viability Assay

IBC cell lines, SUM149PT and SUM190PT, were seeded in 96-well plates and treated with and without Salubrinal in the absence or presence of specific, irreversible caspase inhibitors (caspase-2, -3, -6, -8, -9, and -10) from R&D systems. Enhanced cell survival was determined using the CellTiter-fluor cell viability assay from Promega (#G6080) per the manufacturer’s instructions. This assay measures a conserved and constitutive protease activity within live cells using a fluorogenic substrate. Loss of cell membrane integrity inactivates this protease.

### BrdU ELISA

The effect of Salubrinal treatment on SUM149PT cell proliferation was determined by using a BrdU Cell Proliferation ELISA (#6813) kit (Cell Signaling Technology). This technique is based on the incorporation of the pyrimidine analog BrdU into the DNA of proliferating cells. After its incorporation into DNA, BrdU is detected by immunoassay. Briefly, untreated Salubrinal treated, and NAC and Salubrinal treated cells were cultured 48h. After Salubrinal treatment, cells were pulsed with BrdU for 4 h. The ELISA was performed in triplicate and the absorbance was read at 450 nm.

### OPG ELISA

The conditioned media of untreated or Salubrinal treated SUM149PT and SUM190PT cells were collected, centrifuged, and OPG levels were measured in the supernatants were measured by ELISA (Raybiotech, Peachtree Corners, GA) according to the manufacturer’s instructions. Results are expressed as the amount of OPG secreted (pg/ml) per 10^6^ cells (14).

### DCFDA/H2DCFDA Cellular ROS Assay

DCFDA/H2DCFDA (2’,7’–dichlorofluorescin diacetate, also known as H2DCFDA) is a fluorogenic dye that measures hydroxyl, peroxyl, and other reactive oxygen species (ROS) activity within the cell. DCFDA/H2DCFDA is a cell-permeant reagent and cellular ROS assay from Abcam (#ab113851) quantitatively assess reactive oxygen species in live cell samples. Briefly, untreated Salubrinal treated, and NAC (5 mM), and Salubrinal treated cells were cultured 48h. Cells were collected and stained (30 min at 37°C) with oxidative-sensitive dye DCFDA/H2DCFDA and analyzed immediately with a flowcytometer as per the manufacturer’s instructions. Exogenous H_2_O_2_ is frequently used as a representative ROS in modeling and inducing oxidative stress.

### Statistical Analysis

Differences between samples were analyzed with the Student’s t-test. The statistical significance (t-test) was conducted with respect to untreated cells. Significant differences at *P* < 0.05, 0.01, 0.005, and 0.001 between conditions are indicated by *, **, ***, and **** respectively. All calculations were performed using the GraphPad PRISM version 4.00 for Windows (GraphPad Software, La Jolla, CA, USA).

## Results

### OPG interacts With Major ER Chaperone Protein GRP78/BiP in IBC Cells

**Figure 3:**
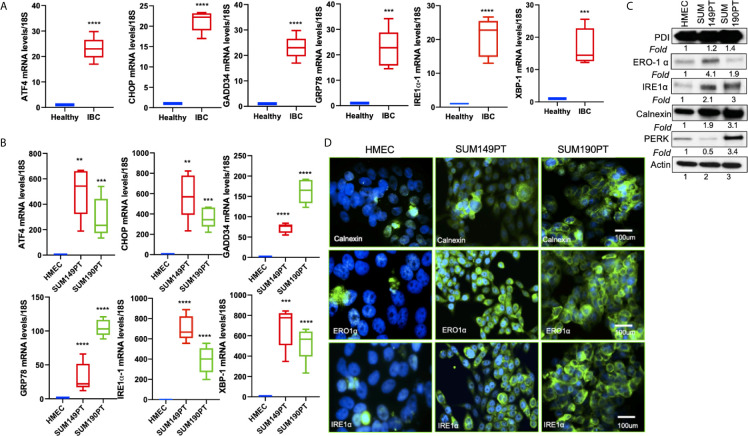
ER stress genes are abundantly expressed in IBC tissue sections and IBC cell lines. **(A)** RNA isolated from healthy and IBC tumor tissue was prepared, converted to cDNA, and gene expression was quantified by real-time RT-PCR using specific primers for ER stress markers as indicated. Each data point represents the average gene expression from six IBC and six healthy control samples. Each point represents the average ± the standard deviation of three experiments. (***) p<0.005, (****) p<0.001 indicates a statistically significant difference compared with healthy tissue. Each reaction was done in triplicate. **(B)** RNA isolated from HMEC, SUM149PT, and SUM190PT cells was converted to cDNA, and gene expression was quantified by real-time RT-PCR using specific primers for ATF4, CHOP, GADD34, GRP78, IRE1α, and XBP-1. Each point represents the average ± the standard deviation of three experiments. (**) p<0.01, (***) p<0.005, (****) p<0.001 indicate a statistically significant difference compared with HMEC cells. Each reaction was done in triplicate. **(C)** ER stress genes proteins are abundantly expressed in IBC cell lines. Lysates prepared from HMEC, SUM149PT, and SUM190PT cells, were tested for protein levels of PERK, IRE1α, calnexin, ERO1α, and PDI. Blots were reprobed with anti-β-actin antibody as a loading control for normalization. Fold expression of each protein was calculated by considering the expression of the protein in HMEC as 1. **(D)** Immunostaining of HMEC and SUM149PT cells seeded in eight-well chamber slides. Cells were fixed, permeabilized, and then stained with primary monoclonal antibodies against ER stress markers, including calnexin, ERO1α, and IRE1α. Cells were developed with Alexa-488 coupled secondary antibody (green). Nuclei were visualized using DAPI as the counterstain (blue).

Previous studies from our lab discovered OPG’s role as an essential paracrine factor involved in reprogramming healthy cells into tumor cells and provided novel mechanisms *via* which OPG activates the downstream signaling pathways driving cell proliferation, cell cycle, and aneuploidy (14). We identified the OPG binding proteins in HMEC and IBC cell lines (SUM149PT and SUM1315MO2). Cell lysates prepared from different cell types were immunoprecipitated using an anti-OPG antibody, followed by LC-ESI-MS mass spectrometry analysis (12). Seventeen bands were selected for the study, and proteins were identified with a confidence range of 99.1% to 68.2% (12). OPG bound to several cellular chaperones such as heat shock 70kDa protein (mortalin), GRP78/BiP, heat shock 60kDa protein (chaperonin), and heat shock gp96 precursor, HSP90AB1 protein, and stress-70 protein (12). To understand whether IBC cells are addicted to OPG (12) and use OPG as a critical survival factor by interacting with master ER stress regulator GRP78/BiP (41), we performed IHC, real-time qPCR, Western blotting, and immunofluorescence assay (IFA) to compare the expression of ER stress proteins in healthy human breast tissue as compared to IBC tumor tissue obtained from patients (**Figures 1**–**3**).

Breast tissue sections were analyzed by comparing chaperone GRP78/BiP expression in healthy tissue and IBC tumor from patients (**Figure 1A**). IBC tissue sections (**Figure 1A**; Ab, Ae, Af) showed upregulation of the target cellular chaperone in addition to increased proliferation and pleomorphism compared to the healthy tissue (**Figure 1A**; Aa, Ac, Ad). BiP/GRP78 protein staining was further validated by immunofluorescence assay using BiP/GRP78 antibody in healthy and IBC tissue (**Figure 1B**). We observed heavy staining for BiP/GRP78 around vessels in the IBC tissue section compared to healthy tissue (**Figure 1B**).

A Proximity ligation assay (PLA) and co-immunoprecipitation were used to evaluate OPG and GRP78/BiP interaction. PLA with primary antibodies against OPG and GRP78/BiP was used to detect OPG and GRP78/BiP interaction (**Figure 2A**). PLA is a highly sensitive and specific technique capable of identifying protein-protein interactions within close-proximity (<40 nm). OPG and GRP78/BiP interaction were visualized as red dots in both IBC cell lines, SUM149PT and SUM190PT (**Figure 2A**). The lack of detection of any PLA signals when either primary antibody was used alone validated the antibody specificity and observed interaction (data not shown).

OPG and GRP78/BiP association were confirmed by co-immunoprecipitation analysis (**Figure 2B**; i and ii). The OPG-GRP78/BiP complex was isolated using an anti-OPG antibody, and then the complex was visualized *via* Western blot analysis using an anti-GRP78 antibody. The OPG-GRP78 complex was seen in IBC cell lines (SUM149PT and SUM190PT) (**Figure 2B**; i and ii). This observed interaction’s specificity was confirmed by an immunoprecipitation reaction using an anti-IgG antibody (**Figure 2B**; ii). Input for the total lysate is shown for OPG and BiP (**Figure 2B**; iii).

Tissue sections obtained from IBC patients also showed upregulation of ER stress markers, including ATF4, CHOP, GADD34, GRP78/BiP, IRE1α, and XBP-1 as compared to the healthy tissue obtained from healthy controls (**Figure 3A**). Compared to the healthy control, HMEC cells, increased expression of ER stress markers was observed in inflammatory breast cancer cell lines SUM149PT and SUM190PT. Real-time PCR results demonstrated 10- to 1000- fold upregulation of ER stress-associated genes in IBC cell lines compared to HMEC controls (**Figure 3B**). These results were corroborated by Western blot analysis (**Figure 3C**) and immunofluorescence (**Figure 3D**). Western blot analysis demonstrated the upregulation of ER stress-associated proteins in IBC cells, albeit not always consistent between the cell lines (**Figure 3C**). Overall, these results support the premise that ER stress is an inherent IBC biology attribute. Immunofluorescence staining showed abundant expression of calnexin, ERO1α, and IRE1α in IBC cell lines SUM149PT and SUM190PT compared to healthy control, HMEC cells (**Figure 3D**).

### Salubrinal Exhibits Cytotoxicity Against IBC Cancer Cells

**Figure 4:**
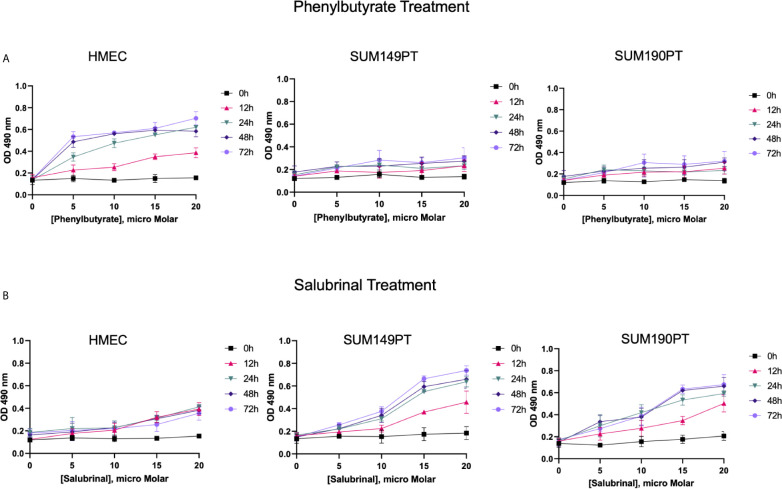
IBC cell lines were chemosensitive to Saburinal treatment. **(A)** Effect of Phenylbutyrate treatment on cytotoxicity. HMEC, SUM149PT, and SUM190PT cells were untreated or treated with Phenylbutyrate at varying concentrations for different time periods, as indicated. Supernatants were collected from the cells to measure the amount of LDH released using spectrophotometry at 490 nm. Each point represents the average ± the standard deviation of three experiments. **(B)** Effect of Salubrinal treatment on cytotoxicity. HMEC, SUM149PT, and SUM190PT cells were untreated or treated with Salubrinal at varying concentrations for different time points, as indicated. Supernatants were collected from the cells to measure the amount of LDH released using spectrophotometry at 490 nm. Each point represents the average ± the standard deviation of three experiments.

Salubrinal and phenylbutyrate have been shown to modulate ER stress pathways (42, 43), and we tested their effect on healthy control cells (HMEC) and IBC cancer cell lines (SUM149PT and SUM190PT) (**Figure 4**). The IBC cell lines and HMEC cells were treated with varying Phenylbutyrate concentrations (**Figure 4A**) or Salubrinal (**Figure 4B**) for different time intervals to select a suitable drug concentration and time interval for subsequent experiments. Cellular cytotoxicity was evaluated using a lactate dehydrogenase (LDH) cytotoxicity assay, which uses spectrophotometry to measure the amount of LDH released from damaged cells. Phenylbutyrate was extremely cytotoxic to healthy control cells (HMEC) than IBC cell lines (SUM149PT and SUM190PT), which designated Phenylbutyrate as a poor candidate for further study (**Figure 4A**). In contrast, Salubrinal was highly cytotoxic to IBC cell lines (SUM149PT and SUM190PT) with minimal effect on the HMEC control cells’ viability at 10μM (**Figure 4B**). We also tested the effect of Salubrinal and Phenylbutyrate on HMEC, SUM149PT and SUM190PT cells and identified the cytotoxic concentration by assessing cell membrane integrity using trypan blue staining and counting live/dead cells (data not shown). Consequently, 10μM Salubrinal was selected for future experiments.

### Salubrinal Regulates ER Stress Pathway Players at Their Gene Expression and Protein Level

**Figure 5:**
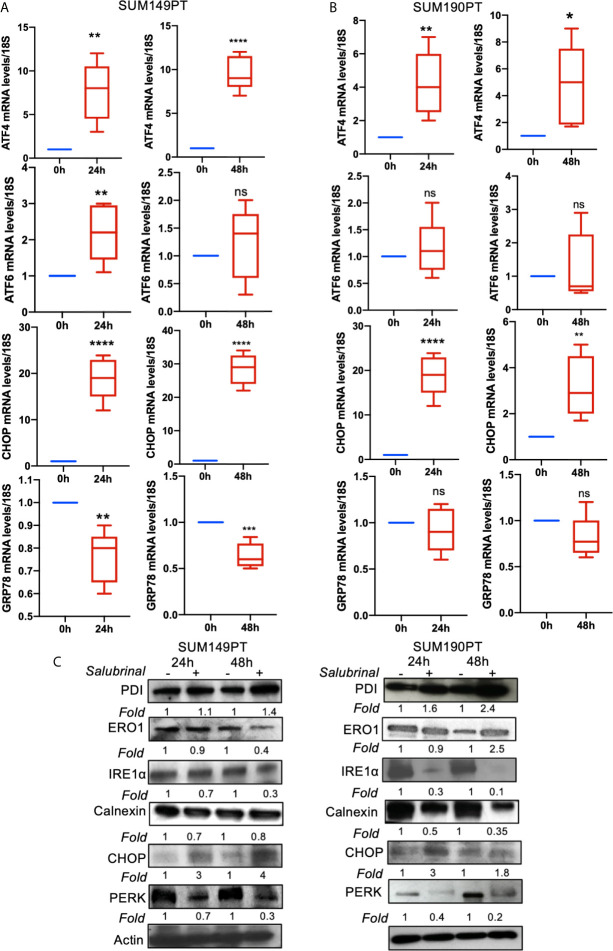
Effect of Salubrinal treatment on ER stress gene expression levels in IBC cell lines. IBC cell lines **(A)** SUM149PT and **(B)** SUM190PT were cultured with or without 10μM Salubrinal for 24 and 48 hours. RNA was prepared from SUM149PT and SUM190PT cells. RNA was converted to cDNA, and ER stress markers ATF4, ATF6, CHOP, and GRP78 were quantitated using real-time PCR, as mentioned in the methods section. Each point represents the average ± the standard deviation of three experiments. (*), p<0.05, (**) p<0.01, (***) p<0.005, (****) p<0.001 indicate a statistically significant difference compared with cells treated for 0h. ns, not significant. **(C)** Effect of Salubrinal treatment on ER protein levels in IBC cell lines. IBC cell lines SUM149PT and SUM190PT were cultured with or without 10μM Salubrinal for 24 and 48 hours. Lysates prepared from SUM149PT and SUM190PT cells were tested for protein levels of ER stress markers ERO1α, PERK, PDI, CHOP, IRE1α calnexin using Western blot analysis. Blots were reprobed with anti-β-actin antibody as a loading control.

To determine Salubrinal treatment’s effect on the ER stress pathway, IBC (SUM149PT and SUM190PT) cell lines were grown with 10μM Salubrinal for 24h and 48h (**Figure 5**). Quantitative RT-PCR and Western blot analysis were used to evaluate ATF4, ATF6, CHOP, and GRP78 gene expression (**Figures 5A, B**) and protein levels (**Figure 5C**).

Salubrinal treatment for 24h or 48h significantly induced ATF4 and CHOP gene expression in SUM149PT and SUM190PT cells (**Figures 5A, B**). Western blot results showed upregulation of CHOP, ERO1, and PDI and downregulation of calnexin in SUM149PT and SUM190PT (**Figure 5C**). However, changes in ER stress markers were variable between cell lines. Salubrinal treatment-induced ERO1 levels in SUM149PT more than SUM190PT cells (**Figure 5C**). IRE1α levels were reduced in SUM190PT more than SUM149PT cells (**Figure 5C**). Salubrinal treatment reduced PERK levels in SUM190PT more than in SUM149PT cells (**Figure 5C**). Overall, treatment of SUM149PT with Salubrinal induced a more substantial increase in ER stress-related proteins compared to SUM190PT (**Figure 5C**).

### Salubrinal Induces Caspase-3-Mediated Apoptosis in IBC Cells

Further characterization of the Salubrinal induced cell death mechanism was sought by analyzing caspases’ activity, a proteases family that plays an essential role in apoptosis. To determine which caspase family member is activated with Salubrinal treatment, we observed the enhanced survival of cells incubated with or without Salubrinal in the absence or presence of specific caspase inhibitors, which included inhibitors of initiator caspases (caspase -2, -8, -9, and -10) and executioner caspases (caspase 3 and 6) (**Supplement Figure 1**). Enhanced survival of SUM149PT and SUM190PT cells was noted with the application of the caspase-3 inhibitor, which indicates a critical role that caspase-3 likely plays in mediating Salubrinal induced cell death in IBC cell lines (**Supplement Figure 1**).

**Figure 6:**
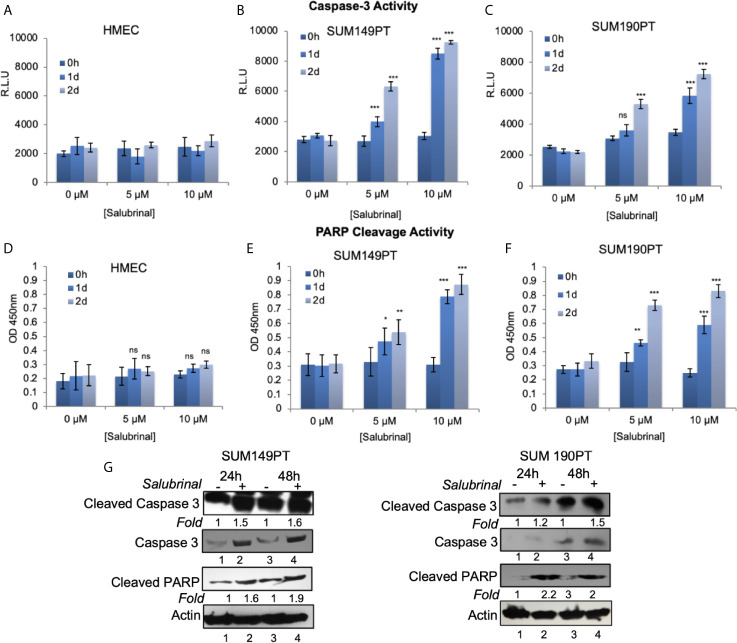
Salubrinal treatment induces caspase-3 activation and PARP cleavage in IBC cells. **(A–C)** HMEC and IBC cell lines, SUM149PT and SUM190PT, were treated with various concentrations of Salubrinal for different time intervals. A luminogenic caspase-3 substrate is added, and the luminescence is measured in relative light units (RLU) as an index for caspase-3 activity. Each reaction was done in triplicate, and each bar represents the mean ± SD for three experiments. **(D–F)** HMEC and IBC cell lines, SUM149PT and SUM190PT, were treated with various concentrations of Salubrinal for different time intervals. Cells were fixed, permeabilized, and incubated with a particular anti-cleaved PARP primary antibody followed by an HRP-labeled secondary antibody. Absorbance (OD) was measured at 450 nm as an index of PARP cleavage. Each reaction was done in triplicate, and each bar represents the mean ± SD for three experiments. (*), p < 0.05, (**) p < 0.01, (***) p < 0.005 indicate a statistically significant difference compared with cells treated for 0h. ns, not significant. **(G)** SUM149PT and SUM190PT cells were cultured with or without 10M Salubrinal for 24 and 48 hours. Lysates prepared and tested for caspase-3, cleaved caspase-3 and cleaved PARP as indicated by Western blot analysis. Blots were reprobed with anti--actin antibody as a loading control. The level of proteins in untreated samples was considered one for fold activation or down-regulation using the quantification method as described in the *Methods* section.

Caspase 3 is processed into cleaved caspase 3 in the early steps of apoptosis and its level positively correlates with the rate of cell death. In cells grown with and without Salubrinal, we analyzed the activation of caspase-3 and the proteolytic cleavage of PARP, a DNA repair enzyme the proteolytic target of caspase-3. Treatment of IBC cell lines SUM149PT and SUM190PT with Salubrinal induced caspase-3 activity with time (**Figures 6B, C**) as well as cleavage of PARP (**Figures 6E, F**), clear indications of apoptosis. Treatment of HMEC cells with Salubrinal did not increase caspase-3 activity with time (**Figure 6A**), as well as cleavage of PARP (**Figure 6D**), clear indications of apoptosis. Salubrinal treatment-induced cleaved caspase-3 and cleaved PARP protein levels in IBC cell lines SUM149PT and SUM190PT cell lysates by Western blotting (**Figure 6G**), which further validated the ELISA results (**Figures 6A–F**).

Salubrinal treatment significantly reduced OPG secretion from SUM149PT and SUM190PT cells as tested by OPG ELISA (data not shown). PARP cleavage was blocked by co-treatment with the broad-range irreversible pan-caspase inhibitor Z-VAD-FMK (inhibits caspase processing and apoptosis induction), suggesting that a caspase cascade mediates Salubrinal-induced apoptosis in IBC cells.

**Figure 7:**
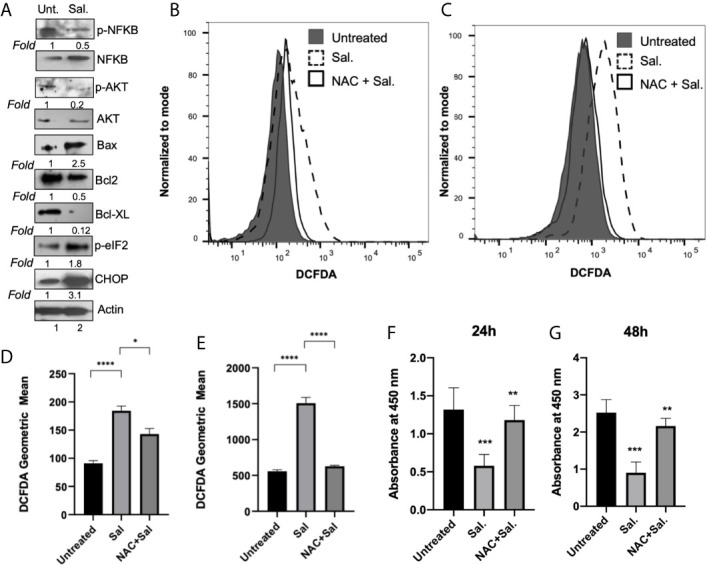
Salubrinal treatment induces cell death potentially by induction of ROS in IBC cells. **(A)** SUM149PT cells were cultured with or without 10μM Salubrinal for 48 hours. Lysates prepared and tested for various inflammation, proliferation, and apoptosis-specific proteins as indicated by Western blot analysis. Blots were reprobed with anti-β-actin antibody as a loading control. p-NFκB and p-AKT were normalized with respect to total NFκB and AKT, respectively. The level of proteins in untreated samples was considered one for fold activation or down-regulation using the quantitation method as described in the Methods section. **(B)** ROS analysis in SUM149PT cells (untreated, Salubrinal treated or NAC and Salubrinal treated) for 48h was done. Raw data of DCFDA fluorescence peaks of a representative experiment. **(C)** ROS analysis in SUM149PT cells (untreated, Salubrinal treated or NAC and Salubrinal treated) for 48h was done. Cells were pulsed with H_2_O_2_ right before analyses in the flowcytometer. Raw data of DCFDA fluorescence peaks of a representative experiment. **(D, E)** Quantification of ROS levels from **(B, E)**, respectively. Each reaction was done in triplicate, and each bar represents the geometric mean ± SD for three experiments. (*), p<0.05 and (****) p<0.001 indicate a statistically significant difference between either untreated and Salubrinal treated or Salubrinal treated and NAC and Salubrinal cotreatment. **(F, G)** Cell proliferation assay in SUM149PT cells (untreated, Salubrinal treated or NAC and Salubrinal treated) for 48h was measured by BrdU assay as described in the methods section. Each reaction was done in triplicate, and each bar represents the absorbance mean ± SD for three experiments. (**), p<0.01 and (***) p<0.005 indicate a statistically significant difference between either untreated and Salubrinal treated or Salubrinal treated and NAC and Salubrinal cotreatment.

We next examined the effect of Salubrinal treatment on major cell survival protein p-Akt and inflammatory protein p-NFκB (**Figure 7A**). Compared to untreated cells, 48 h Salutrinal treatment down-regulated p-NFκB by about 50% and p-Akt by about 80% (**Figure 7A**). At 48h Salubrinal post-treatment, we observed dramatic upregulation of pro-apoptotic Bax, and downregulation of anti-apoptotic Bcl-2, and Bcl-xL (**Figure 7A**). Next, we detected the effect of Salubrinal treatment on eIF2*α* phosphorylation and ER stress pro-apoptotic protein CHOP (**Figure 7A**). Compared to untreated cells, 48 h Salutrinal treatment up-regulated eIF2*α* phosphorylation by about 2-fold and CHOP by about 3-fold (**Figure 7A**).

### Salubrinal Induces ROS-Mediated Downregulation of Cell Proliferation in IBC Cells

To determine whether Salubrinal treatment had any effect on ROS level in IBC cells, we performed a cellular ROS assay using oxidative stress-sensitive probe DCFDA (**Figures 7B, D**). Compared to untreated cells, 48 h Salutrinal treatment significantly up-regulated cellular ROS level (**Figures 7B, D**). Cotreatment with antioxidant NAC and Salubrinal exhibited downregulated ROS levels (**Figures 7B, D**). Similar results (**Figures 7C, E**) were obtained in the experiments where exogenous H_2_O_2_ was used as a representative ROS inducer.

Interestingly, ROS is associated with multiple cell signaling pathways, cell proliferation, and cell death, and we observed downregulation of p-AKT, anti-apoptotic proteins Bcl-2, and Bcl-xL upon Salubrinal in IBC cells. Therefore, we determined whether Salubrinal treatment mediated down-regulation of IBC cell proliferation is mediated *via* ROS using BrdU cell proliferation assay (**Figures 7F, G**). Compared to untreated cells, Salutrinal treatment significantly down-regulated BrdU incorporation during DNA synthesis measured quantitatively as absorbance (**Figures 7F, G**). Cotreatment with antioxidant NAC and Salubrinal exhibited up-regulated BrdU incorporation, an indicator of cell proliferation (**Figures 7F, G**).

## Discussion

IBC presents at a locally advanced or metastatic stage with a poor prognosis. The current standard of care and improved survival rates require advancement in screening and state-of-the-art treatment modalities. Activation of the ER stress response or UPR is associated with numerous fatalities such as the progression of B cell chronic lymphocytic leukemia (CLL) (44, 45), multiple myeloma, and cancers of the breast, prostate, pancreas, and liver (44–46). This hallmark has been exploited with bortezomib development, a proteasome inhibitor used in patients with multiple myeloma. Since the ER stress pathway is a recognized target for therapeutic intervention in cancer, we aimed to understand ER stress and UPR in IBC in context with its unique OPG rich microenvironment (14). OPG is a soluble decoy receptor for tumor necrosis factor receptor (TNF)-related apoptosis-inducing ligand (TRAIL). The rationale for selecting to study UPR has based on 1) our findings in the previous study, where we identified that OPG binds to cellular chaperones including mortalin, chaperonin, BiP protein, HSP90AB1 protein, and heat shock protein gp96 in IBC cells (12) and 2) significantly high expression of ER stress proteins and UPR sensors in IBC cell lines and IBC tumors as compared to their healthy counterparts (**Figures 1**–**3**). Here, we validated the interaction between OPG and an ER chaperone protein critical for protein quality control of the ER called GRP78/BiP (**Figure 2**). These results demonstrate that an OPG rich IBC tumor microenvironment is involved in tweaking the cellular protein homeostasis and driving ER stress in IBC cells. GRP78/BiP has been reported as a prosurvival factor for cells undergoing ER stress. GRP78/BiP directly interacts with apoptotic pathway intermediates, blocks caspase activation, and eventually leads to apoptosis inhibition (47, 48) and increased cell survival (41). GRP78 is also implicated in promoting drug resistance in cancers and regulating angiogenesis (41), an essential hallmark of IBC. We speculate that the OPG-GRP78 interaction in IBC cells cooperatively works to enhance the growth, survival, and spread of IBC.

To determine the effect of the ER stress modulators in IBC cells, we chose Salubrinal and Phenylbutyrate. Salubrinal is a cell-permeable selective inhibitor of eIF2α dephosphorylation that has been associated with up-regulation of ER stress-related cell apoptosis and oxidative stress (49). Salubrinal was preferentially cytotoxic to IBC cell lines, while Phenylbutyrate was cytotoxic to the healthy control cells (**Figure 4**). Importantly, Salubrinal demonstrated minimal toxicity to control HMEC cells (**Figure 4**). Choice of Salubrinal does not undermine the therapeutic potential of Phenylbutyrate, a histone deacetylase inhibitor, approved for the treatment of urea cycle disorders, cancer, hemoglobinopathies, motor neuron diseases, and cystic fibrosis clinical trials (50). It possesses a broad spectrum of molecular functions such as antiviral (51), chromatin regulator and modulator of multiple cell cycle, and apoptosis-related genes (50). Phenylbutyrate has been well studied for its potential in prostate cancer alone (52) or in a combination of Phenylbutyrate and 13-cis retinoic acid (53). Phenylbutyrate along with curcumin could attenuate PA-induced increase in CHOP and GRP78 expression and protect H9C2 cardiomyocytes from lipotoxicity-induced cell death (54). The choice of Salubrinal in the current study was completely based on its minimal toxicity to control HMEC cells and selective toxicity to IBC cells at a low dose (**Figure 4**).

Salubrinal treatment of IBC cell lines upregulated ATF4 and CHOP gene expression (**Figure 5**). ERO1α, PDI, and CHOP consistently increased upon Salubrinal treatment of SUM149PT and SUM190PT cell lines (**Figure 5**). However, the trends in the decrease of PERK and IRE1α levels upon Salubrinal treatment were more pronounced in SUM190PT cells when compared to SUM149PT (**Figure 5**). These differences may be due to cell type variations. Upregulation of CHOP, a proapoptotic transcription factor downstream of PERK and ATF4, demonstrates a potential antitumor strategy of Salubrinal in IBC cells (55) (**Figure 5**).

The ER is also a reservoir for intracellular calcium. It requires IRE1α, and little is known about the molecular mechanisms by which excessive ER stress triggers cell death and Ca(2+) dysregulation *via* the IRE1α-dependent signaling pathway (56). The increased cytosolic concentration of Ca(2+) induces mitochondrial production of reactive oxygen species (ROS), resulting in severe mitochondrial fragmentation, depolarization of membrane potential, and subsequent cell death in IRE1α-deficient cells (56). Cross-talk between oxidative and ER stress has also been observed in palmitic acid (PA)-induced H9c2 cardiomyocytes apoptosis leading to lipotoxic cardiomyopathy (57).

Recently, IRE1α’s potential has been implicated in Ca(2+) homeostasis and cell survival during ER stress and revealed the IRE1α-InsP3R pathway in the ER and the redox-dependent apoptotic pathway in the mitochondrion (56). Salubrinal treatment significantly reduced IRE1α levels in SUM190PT cells, especially at 48h of treatment, suggesting that it might be causing Ca(2+) dysregulation induced mitochondrial abnormalities and eventually cell death in these cells. ERO1*α* has been shown to mediate ER luminal hyper oxidation, leading to calcium leakage and autophagy and mitochondria-mediated apoptosis (58). Salubrinal cytotoxicity in IBC cell lines is associated with activation caspase-3, which appears to be a critical mediator of apoptosis in this pathway (**Figure 6** and **Supplement Figure 1**).

Inhibition of ER stress inhibited the inflammatory response by LPS in mouse granulosa cells, thus highlighting the cross-talk between ER stress and inflammation (59). Consistent with the survival (AKT) protein kinase phosphorylation, we observed the downregulation of inflammatory NFkB (p65) signaling upon Salubrinal treatment (**Figure 7A**).

Oxidative protein folding is catalyzed by several multifunctional ER oxidoreductases, including protein disulfide isomerases (PDI) in eukaryotic cells (60). Oxidative protein folding is a vital resource of ROS production in the cell. After accepting electrons from PDI, ERO1 transfers the electrons to molecular oxygen (O_2_) and produces H_2_O_2_, the major ROS created in the ER (61). Although the activation cascade of caspase pathways during the ER stress is still elusive, our results indicate that Salubrinal induced PDI levels in both IBC cells (**Figure 5C**) and induced ROS (**Figure 7**). N-acetyl-cysteine (NAC), an antioxidant, the ROS scavenger, could rescue ROS induction and cell proliferation (**Figure 7**), suggesting ROS’s role in Salubrinal mediated cell death in IBC cells. CHOP also induces oxidative stress and contributes to cell death during ER stress. CHOP leads to protein misfolding and mitochondrion-dependent induction of oxidative stress (62–64). Since we quantitated total cellular ROS, therefore we can only speculate about the mitochondrial ROS induction upon Salubrinal treatment in IBC cells.

**Figure 8:**
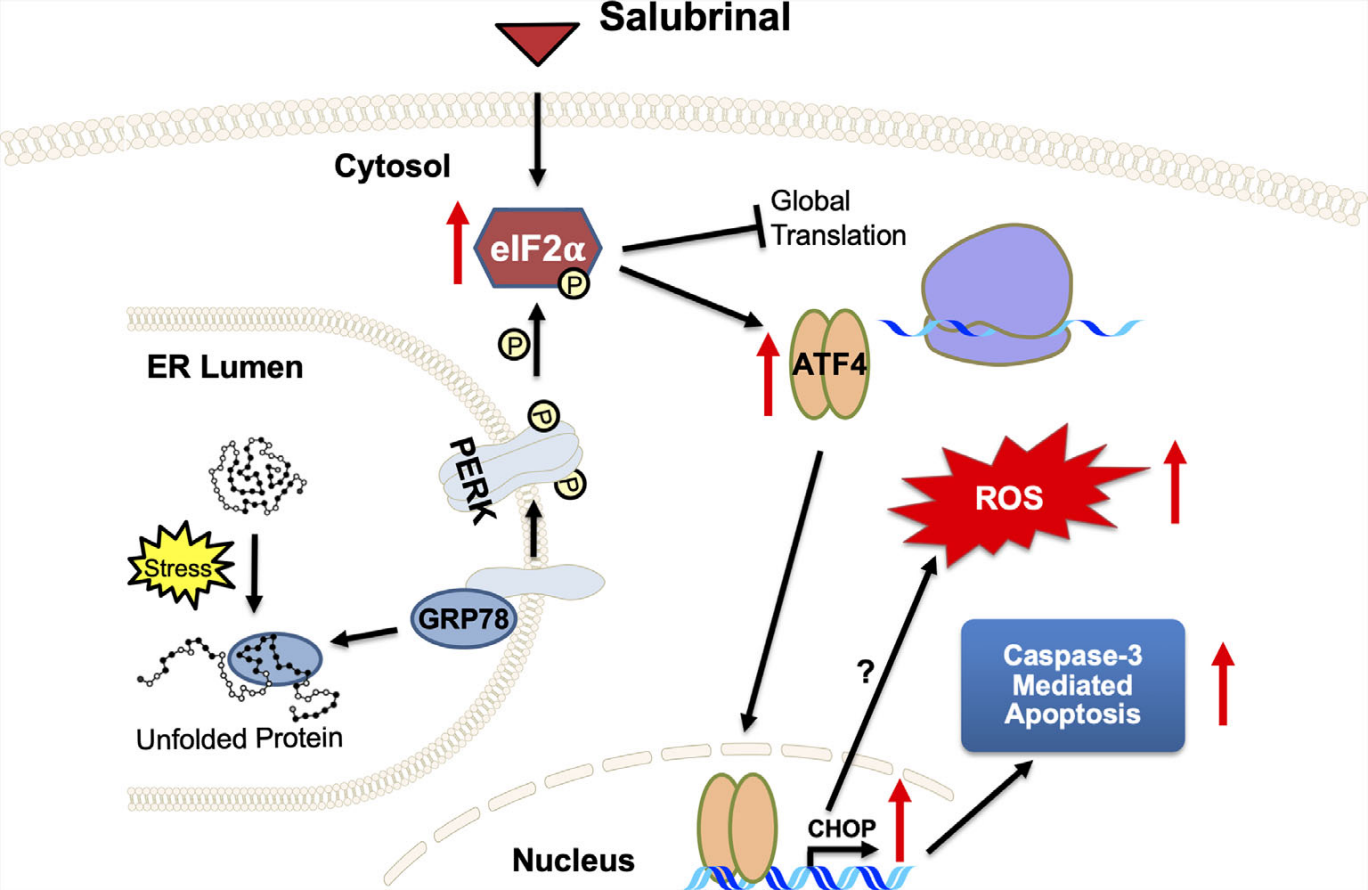
A proposed mechanism of action of ER stress‐prolonging agent Salubrinal on IBC cells. Cellular stress causes unfolded proteins to accumulate in the ER lumen. Chaperone dissociation leads to the activation of the PERK arm of the UPR. PERK is a transmembrane protein that serves as an ER stress sensor and activator of the UPR. PERK phosphorylates eIF2α, thereby inhibiting global translation and selectively promoting ATF4 and CHOP translation, stress-inducible transcription factors. Salubrinal, an eIF2α phosphatase inhibitor, activates the PERK arm of the UPR. Upregulation of CHOP, a proapoptotic transcription factor downstream of PERK and ATF4, demonstrates a potential antitumor strategy of Salubrinal in IBC cells. Salubrinal-treated IBC cells are driven to caspase-3 mediated cell death. Salubrinal treatment-induced cellular ROS and downregulated IBC cell proliferation, which could be rescued upon treatment with antioxidant along with Salubrinal. There may be CHOP-mediated induction of ROS or ROS is the outcome of upregulated oxidoreductases and protein oxidation in the IBC cells.

Salubrinal has been shown to cause TRAIL-induced PARP cleavage through eIF2*α* phosphorylation in hepatoma cells (65). Our results support Salubrinal as a potential targeted therapy for the ER stress pathway in IBC cells (**Figure 8**), which may be beneficial as an adjuvant to standard chemotherapy for breast cancer or maintenance therapy after induction chemotherapy. As described in the introduction, the ultimate effect of Salubrinal on cell survival is multifactorial and depends on the state of the targeted cells. Salubrinal has been shown to have both cytoprotective and cytotoxic impacts when applied to different cells (31, 36, 66, 67). Salubrinal treatment significantly inhibited OPG secretion from IBC cell lines, opening up new avenues for follow-up. It would be interesting to decipher if Salubrinal treatment disrupts the association of OPG-BiP/GRP78 and secretion of RANKL levels in IBC cells. At this point, we do not know whether OPG drives the expression of anti-apoptotic GRP78/BiP. Many studies have supported Paclitaxel as an effective chemotherapeutic modality for IBC (68–70). Paclitaxel appears to be one of the most promising antineoplastic agents of the last decade, with demonstrated activity in advanced and refractory ovarian, breast, lung, and head and neck cancers. Given this proposed anti-proliferative mechanism and safety profile, Salubrinal may be ideal to be tested in combination treatment with Paclitaxel *in vitro* in IBC cell lines. Salubrinal mediated upregulation of eIF2α phosphorylation could increase doxorubicin sensitivity in MCF-7-driven cells that have acquired resistance to doxorubicin (MCF-7/ADR) (36). Salubrinal and rapamycin combination demonstrated antitumor effects in a highly aggressive tumor called cholangiocarcinoma (37). An exquisite study extensively studied the adaptive redox mechanisms of SUM149 cells, and it was demonstrated these cells become resistant to chemo- and radio- therapeutics (71). This resistance is because they lose their ability to accumulate ROS, which results in apoptosis inhibition and enhances cancer cell survival (71). Given the fact that Salubrinal treatment induces ROS in SUM149PT (**Figure 8**) makes it a good drug of choice for combination therapy to regulate their redox status (71). Our study has some limitations as it is focused on few cell lines, utilizes a limited set of human tissue sections, and lacks *in vivo* testing. Still, it opens up new avenues for Salubrinal to be tested in IBC. It is a proof-of-concept study for Salubrinal’s potential as a novel therapeutic intervention in IBC cells that demands further investigation.

## Data Availability Statement

The raw data supporting the conclusions of this article will be made available by the authors, without undue reservation.

## Ethics Statement

The studies involving human participants were reviewed and approved by Advocate Lutheran General Hospital under the approved IRB (IRB00001341). The patients/participants provided their written informed consent to participate in this study.

## Author Contributions

NS-W: Conception and design, AA, KA, OP, MR, SG, and AD: acquisition of data, analysis, and interpretation. NS-W, AA and KA: interpretation of data and writing of the manuscript. HM provided human samples. All authors contributed to the article and approved the submitted version. 

## Funding

We are grateful for funding support from the Center for Cancer Cell Biology, Immunology and Infection, RFUMS-Advocate Lutheran General Hospital grant and RFUMS start-up fund to NS-W. The funders had no role in the design, decision to publish, or preparation of the manuscript.

## Conflict of Interest

The authors declare that the research was conducted in the absence of any commercial or financial relationships that could be construed as a potential conflict of interest.

## Supplementary Material

Supplementary Figure 1 Salubrinal treatment induces caspase-3 mediated cell death in IBC cells. IBC cell lines SUM149PT and SUM190PT were treated with and without Salubrinal in the absence or presence of specific, irreversible caspase inhibitors (caspase -2, -3, -6, -8, -9, and -10). The fluorogenic substrate GF-AFC was added, and fluorescence was measured in relative fluorescence units (RFU) as an index of viability. Each reaction was done in triplicate, and each bar represents the mean ± SD for three experiments. *p<0.05, **p<0.01, ***p<0.005, ****p<0.001 indicate a statistically significant difference compared with respective untreated cells. ns, not significant.

## Abbreviations

ATF4, activating transcription factor 4; CHOP, C/EBP homologous protein; GADD153, growth arrest and DNA damage-inducible gene 153; eIF2α, eukaryotic initiation factor 2 (alpha-subunit); ER, endoplasmic reticulum; ERO1, ER oxidoreductase 1; GRP78, glucose-regulated protein (78 kDa); GADD34, growth arrest and DNA damage-inducible protein 34; HER-2, human epidermal growth factor receptor 2; HMEC, human mammary epithelial cells; IBC, inflammatory breast cancer; IRE1α, Inositol-requiring enzyme 1; PARP, poly-ADP-ribose polymerase; PERK, PKR-like endoplasmic reticulum kinase; EIF2AK3, eukaryotic initiation factor 2 alpha kinase 3; PDI, protein disulfide-isomerase; XBP-1, X-box binding protein 1; TME, tumor microenvironment.
