# Supplementary figures and images for: Salubrinal Exposes Anticancer Properties in Inflammatory Breast Cancer Cells by Manipulating the Endoplasmic Reticulum Stress Pathway
Source: Front Oncol. 2021 May 20;11:654940. doi: 10.3389/fonc.2021.654940 (PMC8173155; doi:10.3389/fonc.2021.654940)

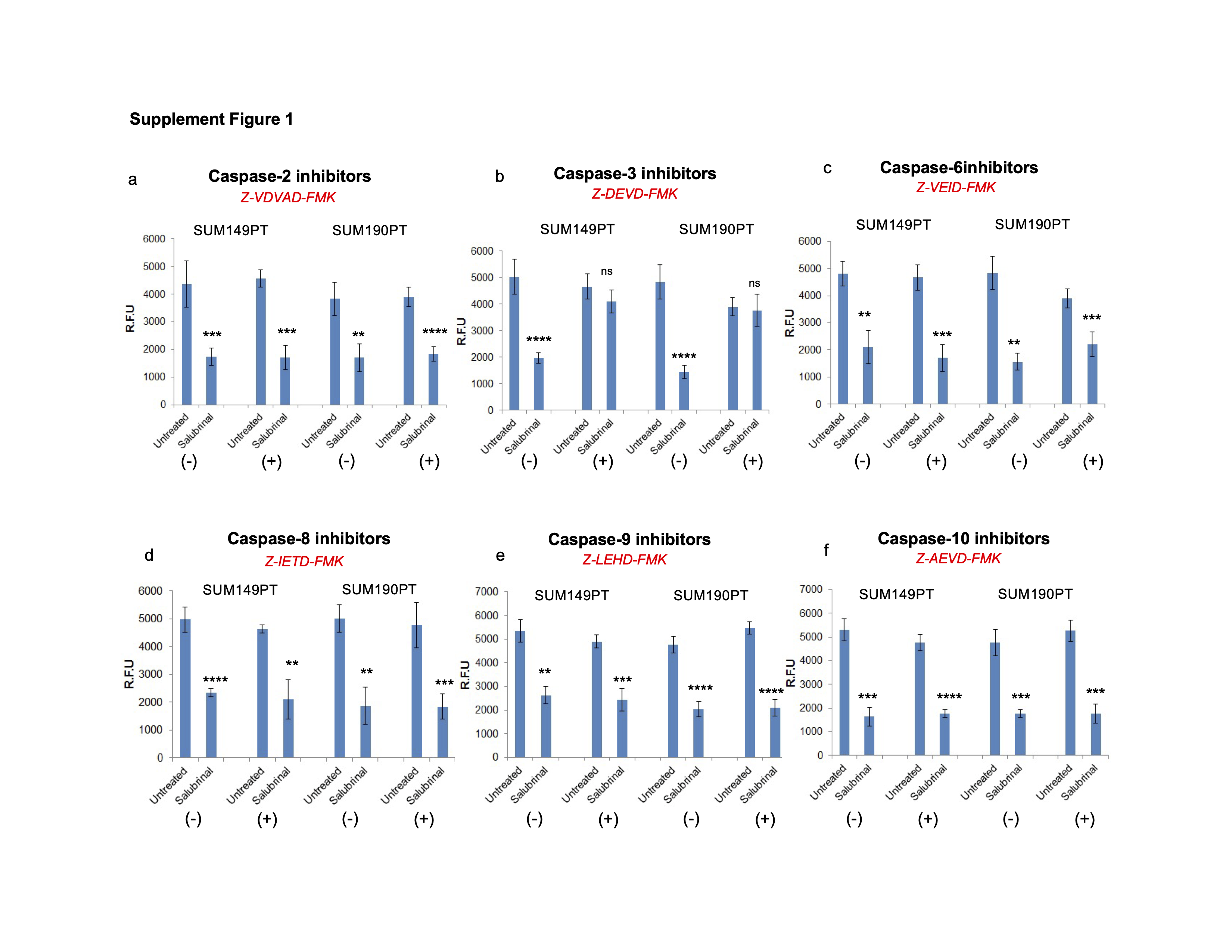

Supplement: Supplementary Figure 1 — Salubrinal treatment induces caspase-3 mediated cell death in IBC cells. IBC cell lines SUM149PT and SUM190PT were treated with and without Salubrinal in the absence or presence of specific, irreversible caspase inhibitors (caspase -2, -3, -6, -8, -9, and -10). The fluorogenic substrate GF-AFC was added, and fluorescence was measured in relative fluorescence units (RFU) as an index of viability. Each reaction was done in triplicate, and each bar represents the mean ± SD for three experiments. *p<0.05, **p<0.01, ***p<0.005, ****p<0.001 indicate a statistically significant difference compared with respective untreated cells. ns, not significant. [file Image_1.tiff]
